# New Trends in Impedimetric Biosensors for the Detection of Foodborne Pathogenic Bacteria

**DOI:** 10.3390/s120303449

**Published:** 2012-03-12

**Authors:** Yixian Wang, Zunzhong Ye, Yibin Ying

**Affiliations:** College of Biosystems Engineering and Food Science, Zhejiang University, Hangzhou 310058, China; E-Mails: wang_yi_xian1986@sina.com (Y.W.); zzye@zju.edu.cn (Z.Y.)

**Keywords:** impedimetric biosensors, foodborne pathogenic bacteria, nanomaterials, microfluidics technique

## Abstract

The development of a rapid, sensitive, specific method for the foodborne pathogenic bacteria detection is of great importance to ensure food safety and security. In recent years impedimetric biosensors which integrate biological recognition technology and impedance have gained widespread application in the field of bacteria detection. This paper presents an overview on the progress and application of impedimetric biosensors for detection of foodborne pathogenic bacteria, particularly the new trends in the past few years, including the new specific bio-recognition elements such as bacteriophage and lectin, the use of nanomaterials and microfluidics techniques. The applications of these new materials or techniques have provided unprecedented opportunities for the development of high-performance impedance bacteria biosensors. The significant developments of impedimetric biosensors for bacteria detection in the last five years have been reviewed according to the classification of with or without specific bio-recognition element. In addition, some microfluidics systems, which were used in the construction of impedimetric biosensors to improve analytical performance, are introduced in this review.

## 1. Introduction

In recent years, diseases and productivity losses caused by foodborne pathogenic bacteria have attracted considerable attention. Thousands of foodborne pathogenic bacteria have been found to affect the health and safety of the world’s populations of humans, animals and plants. Among these bacteria, *Campylobacter*, *Salmonella*, *Listeria monocytogenes*, *Escherichia coli* (*E. coli*) O157:H7, *Staphylococcus aureus*, and *Bacillus cereus* are the major foodborne pathogen bacteria, which are responsible for the majority of foodborne illness outbreaks [1–5]. Therefore, it is of great importance to develop methods for foodborne pathogenic bacteria detection.

Several methods have been explored for the bacteria determination, including the culture and colony counting method, polymerase chain reaction (PCR), and immunology-based method [6–10]. The traditional culture and colony counting method has been a practical for the detection and identification of pathogens in food, including microbiological culturing and isolation of the pathogen, followed by confirmation by biochemical and serological tests, which takes up to 5–7 days to get a result [11]. Although it can obtain reliable result, it is labor intensive and time consuming, which cannot satisfy the request for bacteria detection on-the-spot detection. The PCR and enzyme-linked immunosorbent assay (ELISA) are a lot less time-consuming than the traditional culture and colony counting method, which usually takes 30 mins or a few hours to achieve detection result [9,12]. However, there are still key issues that need to be considered in the development of rapid methods for the detection of foodborne pathogens, including differentiation of live and dead cells, automation, cost, simplicity, training, and accuracy.

Impedance technique, as one kind of the electrochemical biosensors, has been proved to be a promising method for foodborne pathogenic bacteria detection due to its portability, rapidity, sensitivity, and more importantly it could be used for on-the-spot detection [13–16]. Generally, the impedance detection techniques can be classified into two types depending on the presence or absence of specific bio-recognition elements. The first type works by measuring the impedance change caused by binding of targets to bioreceptors (antibodies and nucleic acids) immobilized onto the electrode surface, while the detection principle of the second type is based on metabolites produced by bacterial cells as a result of growth. The articles about impedance biosensors for bacteria detection before 2007 have been reviewed comprehensibly [11], however, in the last five years some new trends in this area have emerged, including the use of nanomaterials, microfluidics techniques and new specific bio-recognition elements such as bacteriophage and lectin. The applications of these new materials or techniques have provided unprecedented opportunities for the development of high-performance impedance bacteria biosensors. Nanomaterials in particular have exhibited unique advantages for constructing impedimetric biosensors and there are an abundance of research articles about that topic, so in this paper, we will focus on those new trends in the development of impedance bacteria biosensor. The significant developments of impedimetric biosensors for bacteria detection in the past five years have been reviewed according to the classification of with or without specific bio-recognition element. In addition, some microfluidics systems, which were used in the construction of impedimetric biosensors to improve analytical performance, have been covered in this review.

## 2. Principle of Impedance Technique

Electrical impedance (Z) is defined as the ratio V(t)/I(t) of an incremental change in voltage to the resulting change in current. From this definition, the impedance Z is the quotient of the voltage-time function V(t) and the resulting current−time function I(t):

$$Z=\frac{V(t)}{I(t)}=\frac{1}{Y}=\frac{V_{0}\;\text{sin}(2\pi ft)}{I_{0}\;\text{sin}(2\pi ft+\varphi )}$$

where V_0_ and I_0_ are the maximum voltage and current signals, *f* is the frequency, t is time, φ is the phase shift between the voltage-time and current-time functions, and Y is the complex conductance or admittance. The impedance is a complex value affected by multiple factors, which is described either by the modulus |Z| and the phase shift φ or alternatively by the real part Z_R_ and the imaginary part Z_I_ of the impedance [17].

Electrochemical impedance spectroscopy (EIS) is a method that describes the response of an electrochemical cell to a small amplitude sinusoidal voltage signal as function of frequency [18]. It is an ideal tool for observing the dynamics of biomolecule interactions [19]. The most popular formats for evaluating EIS data are the Nyquist and Bode plots. In the Nyquist plot, the imaginary impedance component (z″) is plotted against the real impedance component (z′). In the Bode plot, both the logarithm of the absolute impedance (|Z|) and the phase shift (φ) are plotted against the logarithm of the excitation frequency.

**Figure 1.**
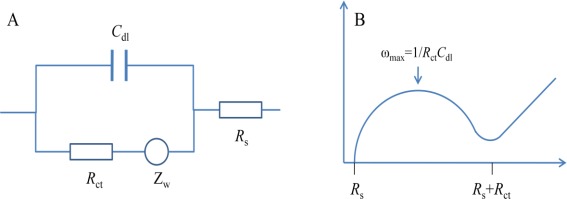
(**A**) the typical Nyquist diagram for the AC impedance measurements; (**B**) the Randle equivalent circuit.

In order to express the characterization of surfaces, layers or membranes after the immobilization of biomolecules and bacteria binding, EIS is often analyzed using an equivalent circuit which is used to curve fit the experimental data and extract the necessary information about the electrical parameters responsible for the impedance change [17]. Since the electrochemical cell is a complex system, more than one circuit model can fit the experimental data [20]. The simplest, and in fact the most frequently used equivalent circuit for modelling the EIS experimental data is the so-called Randles circuit (Figure 1(A)), which comprises the uncompensated resistance of the electrolyte (*R*_s_), in series with the capacitance of the dielectric layer (*C*_dl_), the charge-transfer resistance (*R*_ct_) and the Warburg impedance (*Z*_w_) [18]. In the Nyquist plot shown in Figure 1(B), a typical shape of a Nyquist plot includes a semicircle region lying on the real axis followed by a straight line. The linear part (ψ = π/4), observed at the low frequency range, implies a mass-transfer limited process, whereas the semicircle portion, observed at high frequency range, implies a charge-transfer limited process. From the Nyquist plot, the values for *R*_s_ and *R*_ct_ can be easily determined. The double layer capacitance can be calculated from the frequency at the maximum of the semicircle (*ω* = 2π*f* = 1/*R*_ct_*C*_dl_). The charge-transfer resistance *R*_ct_ and the double layer capacitance *C*_dl_ are the most important electrical parameters in analyzing the impedance signal change for detection of bacteria.

## 3. Types of Impedance Detection Techniques for Foodborne Pathogenic Bacteria Detection

### 3.1. Detection Based on the Use of Specific Bio-Recognition Element

**Figure 2.**
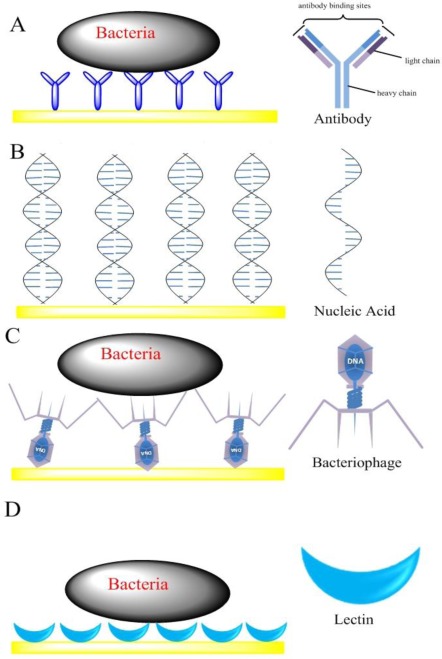
Schematic view of impedimetric biosensors fabricated by different bio-recognition elements: (**A**) Antibody-based sensor; (**B**) Nucleic Acid-based sensor; (**C**) Bacteriophage-based sensor; (**D**) Lectin-based sensor.

Impedimetric biosensors have been designed by immobilizing bioreceptors (such as antibodies, nucleic acids, bacteriophages and lectins) at the surface of a solid electrode. The binding ability of bacteria and the bioreceptors is then verified through the detection of either a shift in impedance, or change in capacitance or admittance at the bulk of the electrode interface due to the insulating properties [21]. The bacterial cell membrane consists of a lipid bilayer, where the lipid molecules are oriented with their polar groups facing outwards into the aqueous environment, and their hydrophobic hydrocarbon chains pointing inwards to form the membrane interior. Pethig reported that natural cell membranes (thickness 5–10 nm) show a membrane capacitance of 0.5–1.3 μF/cm^2^ and a membrane resistance of 10^2^–10^5^ Ω·cm^2^. If bacterial cells attach on an electrode surface, they would effectively reduce the electrode area that the current reaches and hence increases the interface impedance. Here, according to the types of bioreceptors, the impedimetric biosensors were classified into four different categories, including antibody-based sensors, nucleic acid-based sensors, bacteriophage-based sensors and lectin-based sensors (Figure 2).

#### 3.1.1. Antibody Sensors

Impedimetric biosensors based on directly immobilizing antibodies on the surface of an electrode for the detection of bacteria, called impedimetric immunosensors, are constructed by immobilizing antibodies on the electrode surface, and then probing the attachment of the bacterial cells by measuring the change in electrical properties over a range of frequency due to the insulating properties of the cell membrane [11]. Antibodies have long been the most popular bio-recognition elements. The main advantage of the use of antibodies as bio-recognition elements is their sensitivity and selectivity. A wide variety of impedimetric immunosensors reported for different bacteria detection applications exists in the last five years [22,23].

There are some main means for improving impedimetric immunosensors efficiency: (I) improving immobilization methods of antibody on the electrode surface; (II) improving electrode performance to enhance sensitivity; (III) using enzyme-labeled and nanomaterials to amplify detection signal; (IV) optimal equivalent circuit for analyzing impedance change; (V) the dielectrophoresis technique for concentrating samples.

The immobilization method is the key process in the construction of impedimetric biosensors, since the efficiency of antibody immobilization on the electrode surface can profoundly affect the analytical performance of impedance biosensors. There are several methods for the immobilization of antibodies on the electrodes, including physical adsorption, self-assembled monolayer (SAM) and biotin-streptavidin system. Physical adsorption is the simplest and straightforward immobilization method that depends on the non-specific interactions of the biomolecules with the solid substrate. These non-specific interactions contain various non-covalent bridges, such as ionic and hydrogen bonds, hydrophobic interactions, and van der Waals forces. Yang *et al.* [24] developed a label-free electrochemical impedance immunosensor by physical adsorption method to immobilize anti-*E. coli* antibodies onto an indium-tin oxide interdigitated array microelectrode (IDAM) for detection of *E. coli* O157. The equivalent circuit consisted of an ohmic resistor of the electrolyte between two electrodes, double layer capacitor, an electron-transfer resistor, and Warburg impedance around each electrode. Experimental data fitting to the equivalent circuit showed that the electron transfer resistance and electrolyte resistance were responsible for the detection of *E. coli* O157:H7 cells. The detection range of the biosensor was from 4.3 × 10^5^–4.36 × 10^8^ cfu·mL^−1^ with the detection limit of 10^6^ cfu mL^−1^. In spite of its convenience, this method is generally restricted to a limited number of applications due to low stability and the random orientation of the bio-recognition elements immobilizing on electrodes, resulted in the decrease of the binding bacteria. SAM is considered an ideal method to immobilize the antibodies in the construction of impedimetric immunosensors for bacteria detection [18,25–29], which provides a convenient and flexible route to generate ultrathin and ordered biological monomolecular films on a variety of substrates by organic molecules (both aliphatic and aromatic) containing free anchor groups such as thiols, disulphides, amines, silanes, or acids [29,30]. Geng *et al.* [27] used mercaptoacetic acid to form SAM for immobilizing the anti-*E. coli* antibodies on an Au electrode. The immobilization of antibodies on the SAM was carried out through a stable acyl amino ester intermediate generated by EDC and NHS, which can facilitate the formation of a suitable intermediate to condense antibodies on the SAM and enhance the stability sensitivity of the developed immunosensor. A linear relationship between the electron-transfer resistance and the logarithmic value of *E. coli* concentration was found in the range of *E. coli* cells from 3.0 × 10^3^–3.0 × 10^7^ cfu·mL^−1^ with the detection limit of 1.0 × 10^3^ cfu·mL^−1^. However, the SAM immobilization method still suffers from some drawbacks, such as electric field induced and thermal desorption of monolayer and nonspecific adsorption due to high surface energy. Since the biotin-strept(avidin) system has high binding capacity for antibody immobilization due to the high affinity constant between streptavidin and biotin, it is also widely used to immobilize antibodies on solid support interface to construct impedimetric immunosensors. Barreiros *et al.* [25] compared the effect of two different antibody immobilization strategies: one is the use of chemical bond formation between antibody amino groups and a carboxylic acid-containing SAM molecule, and the other is based on linking a biotinylated anti-*E. coli* to avidin on a mixed-SAM. Very low concentrations of *E. coli* (10–100 cfu·mL^−1^) can be detected with the biosensors fabricated by the above design strategies. Though the biotin-strept(avidin) system is an effective method to immobilize the bio-recognition elements on the surface of solid supports, this method has some disadvantages such as the high cost of the reagents involved and the need for a suitable linker layer such as SAM in some cases to attach to the electrode.

Traditionally, three or four macro-sized metal electrodes system is used to measure impedance. With the development of minimization techniques, microelectrodes have been used in fabrication of impedimetric immunosensors due to the miniaturization of the sensor and improvement of the sensitivity [24,31]. Among these techniques, IDAM which has some advantages over the macro-sized electrode, including detecting small amounts of generated electrode products, eliminating the need for a reference electrode, providing simple means for obtaining a steady-state current response, and low response times, has been widely employed to fabricate impedimetric immunosensors [20]. According to the previous research, when the electrode bands become narrower, the biosensor becomes more sensitive. Stephen and coworkers [32] immobilized polyclonal antibodies onto an interdigitated gold electrode array. Each sensor chip had a total of 1,700 electrodes to form a large active area of 9.6 mm^2^. Each electrode finger had a length of 750 μm, a width of 3 μm and an in-between spacing of 4 μm. Each sensor was diced to a dimension of 12 mm × 8 mm. The biosensor was able to discriminate between cellular concentrations of 10^4^–10^7^ cfu·mL^−1^. At present, the commercial company producing IDAM for impedance detection is ABTECH Scientific, Inc.

In addition to minimizing the size of electrodes, some new electrode materials are used to construct impedimetric immunosensors, which enhance the performance of the biosensors for bacteria detection. It is reported that the electrode made from macroporous silicon (3D) structure could be used as the efficient trapping platform for bacteria detection, and the obtained sensitivity was found to be more sensitive than a planar (2D) sensor. Wan *et al.* [28] developed a 3D-immunosensor based on antibody-functionalized 3D-foam Ni substrate as the trapping platform for detection of sulfate-reducing bacteria using impedimetric technique, which can detect the sulfate-reducing bacteria concentration range of 2.1 × 10^1^–2.1 × 10^7^ cfu·mL^−1^.

In order to amplify the detection signal and achieve lower detection limits, the main interaction signal may be enhanced by case-specific amplification schemes, like enzyme-labeled amplification. Ruan *et al.* [33] reported an impedimetric immunosensor for bacteria detection using horseradish peroxide enzyme-labeled for signal amplification. After binding the bacterial cells, secondary antibodies with horseradish peroxide were used to produce precipitation of insoluble products on the electrode surface building thus a rather insulating layer in order to amplify impedance signal due to blocking the electron transfer. A linear response in the *R*_ct_ for the concentration of *E. coli* cells was found between 6 × 10^4^ and 6 × 10^7^ cfu mL^−1^ and the detection limit was 6 × 10^3^ cfu·mL^−1^.

The equivalent circuit used to curve fit the experimental data and extract the necessary information about the electrical parameters responsible for the impedance change is of great importance to analyze the EIS. Some efforts have been devoted into optimize the equivalent circuit. RoyChaudhuri *et al.* [34] developed a biomolecule compatible electrical model to establish a rapid and cost effective method for quantification of antibodies immobilized and bacteria captured which can be applied for the standardization of any new developing technique for improving immobilization and capture efficiency. The model had been applied to retrieve the information about actual number of antibodies immobilized on the electrode surface and the actual concentration range of *E. coli* K12 bacteria captured on the gold surface, which are 15.96 × 10^10^ and 10^6^–10^3^ cfu·mL^−1^, respectively.

**Figure 3.**
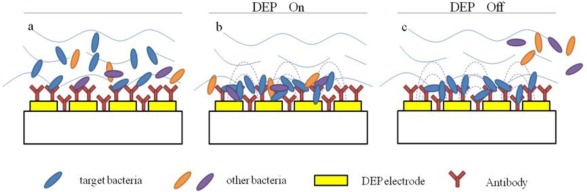
Principle of impedance coupled with dielectrophoresis and electropermeabilization.

Recently, some researchers have combined the dielectrophoretic impedance measurement (DEPIM) technique with impedimetric biosensors for bacteria detection (Figure 3) [35,36]. The DEPIM utilizes the positive dielectrophoretic force to trap suspended biological cells onto the electrode in the form of pearl chains and then measured an impedance signal [37–39], which can detect bacteria in shorter time than traditional impedance methods due to the effect of dielectrophoretic force. Suehiro *et al.* [40] developed a selective detection method for specific bacteria by using a DEPIM method in conjunction with an antigen-antibody reaction. Antibodies were immobilized on the electrode chip before the preliminary bacteria trapping by positive dielectrophoresis (DEP). The bacteria were attracted to the electrode gap under the action of the positive DEP force and finally brought into contact with the glass surface to be bound with the immobilized antibodies. It was also confirmed that the proposed method realized selective detection of the target bacteria from a mixed suspension with non-target bacteria.

#### 3.1.2. Nucleic Acid Sensors

In recent years, nucleic acid analysis has become an important tool for identification of disease-causing microorganisms in food and environment [41–45]. In the case of nucleic acid as bioreceptor for pathogen detection, the identification of a target analyte’s nucleic acid is achieved by matching the complementary base pairs that are often the genetic components of an organism. Since each organism has unique nucleic acid sequences, any self-replicating microorganism can be easily identified [4]. Compared to antibody, the biological recognition layers formed by nucleic acid have many advantages. First, nucleic acids can be chemically synthesized with high purity, avoiding batch-to-batch variation. Second, during synthesis they can be chemically modified with some functional groups, like −HS, −NH_2_, biotin, and so on, which can be easily immobilized onto the electrode surface. Third, the nucleic acid is highly stable and reusable after simple thermal melting of the DNA duplex, which is suitable for biosensor regeneration.

Due to their wide range of physical, chemical and biological activities, nucleic acid based biosensors have been reported by many researchers for the detection of food pathogens [4]. Commonly, nucleic acid based impedimetric biosensors contain immobilized nucleic acid probes that specifically hybridize to their complementary sequences in bacteria samples and an impedance transducer which transforms biomolecule recognition signal into an impedance signal (Figure 2(B)) [16]. Pinar *et al.* [46] developed nucleic acid based impedimetric biosensors for rapid and selective detection of *Bacillus anthracis* (*B. anthracis*). An alkanathiol-linked or unlabeled capture probe related to *B. anthracis* was immobilized onto gold or graphite electrode surface. The extent of hybridization between probe and target sequences was determined by using EIS. EIS analysis was based on *R*_ct_ in the presence of [Fe(CN)_6_]^3−/4−^ and Meldola’s Blue reduction signal as hybridization indicator. The method provided a highly sensitive detection of DNA of 1 × 10^4^ copies (about 1.7 × 10^−20^ mol) of original genomic HBV DNA by combining a PCR procedure.

Although it is undeniable that nucleic acid based impedimetric biosensors have played an increasingly important role in the field of bacteria detection on site applications, they still suffer from some drawbacks. For example, EIS signals resulting from nucleic acid-based impedimetric biosensors are remarkably affected by repulsions between the negatively charged phosphate backbone and redox anions such as [Fe(CN)_6_]^3−/4−^ that make the quantitation of DNA hybridization reactions rather difficult. Moreover, nucleic acid-based methods are unable to discriminate between viable and nonviable cells.

#### 3.1.3. Bacteriophage Sensors

Bacteriophages are viruses, which are made of an outer protein coat that encases genetic material (DNA or RNA) [47]. They can recognize specific sites on the bacterium surface to which they bind and inject genetic material (Figure 2(C)). Since the recognition is highly specific, it can be used for the typing of bacteria [48–50]. Bacteriophages have several desirable advantages for the development of a real-time sensor to rapidly and selectively detect target bacteria in a variety of harsh conditions, such as under acidic or basic pH ranges, and even in the presence of nucleases or proteolytic enzymes. In addition, bacteriophages are not only more cost-effective than antibodies, but also more amenable than antibodies to manipulation at the molecular level to improve their interaction with bacteria [51].

Due to these advantages, the bacteriophages are ideal bioreceptors to make impedimetric biosensors for bacteria detection [52–54]. An example can be found in the determination of *E. coli* bacteria by covalently immobilization of T4 bacteriophages onto functionalized screen-printed carbon electrodes. The *R*_ct_ undergoes a decrease with increasing bacteria concentration ranging from 10^2^ to 10^8^ cfu·mL^−1^, which is contrary to what is usually observed for simple attachment of intact bacteria cells to an electrode surface in impedimetric immunosensors (an increase of *R*_ct_ with increasing concentration of intact bacteria at the surface). Since the lysis of bacteria resulting from the attack of bacteriophage could lead to the release of highly mobile ionic material (such as K^+^ and Na^+^), the conductivity of the media near the electrode surface was increased. Correspondingly, the values related to *R*_ct_ show a clear decrease with increasing concentration of *E. coli* cells. The bacteriophage impedance biosensor showed excellent specificity for target bacteria *E. coli* with a detection limit of 10^4^ cfu mL^−1^, and no significant change in impedance was observed in the presence of *Salmonella* [55]. Gervais *et al.* [56] developed an impedimetric biosensor for *E. coli* detection based bacteriophages immobilized on gold surfaces through biotin/streptavidin system. Such chemical attachment of bacteriophages onto sensor surfaces could in turn be leveraged in highly sensitive and more rapid transduction platforms such as surface plasmon resonance (SPR), quartz crystal microblance (QCM), and microcantilevers. Webster *et al.* [57] developed an impedimetric microelectrode array biosensor based bacteriophage for the detection of bacteria. The results indicated that reducing the width and gap of electrode and using the working solution with lower relative dielectric permittivity can increase the sensitivity of impedimetric biosensors for pathogenic bacteria.

#### 3.1.4. Lectin Sensors

More recently, the use of lectin as the bioreceptor in biosensors has been proven to be very promising and effective. Lectins are plant or animal proteins or glycoproteins, which can bind selectively and reversibly with mono- and oligosaccharide components of polysaccharide structures that are major structural components of bacterial cells surfaces. Recognition of these carbohydrates on the surface of bacteria can be used for specific identification of target bacteria [58,59]. Such a recognition system is superior to antibody or nucleic acid based systems, since the latter systems always require a prior knowledge on the target and specific reagents, which become increasingly problematic when the identities of which are unknown [60]. Furthermore, the molecule size of lectins are much smaller than antibodies, thus they allow higher densities of carbohydrate-sensing elements leading to higher sensitivity and lower non-specific adsorption [61,62]. Finally, agglutination between the lectins and bacteria occurs quickly. Gamella *et al.* [61] reported the lectin modified screen-printed gold electrodes for the impedimetric label-free detection of *E. coli* bacteria. The biotinylated lectins were immobilized on the gold electrode, and then the selectively binding between bacteria and lectins was determined by EIS. The impedance biosensor showed a good performance with a detection range of 5.0 × 10^3^ and 5.0 × 10^7^ cfu·mL^−1^. A similar approach was used to detect sulfate-reducing bacteria by Wan *et al.* [63]. The lectin-concanavalin A as the bioreceptor was assembled on the gold electrode with 11-mercaptoundecanoic acid to bind sulfate-reducing bacteria. The lectin-based impedance biosensor exhibited good performance for sulfate-reducing bacteria detection with a concentration range of 1.8 to 1.8 × 10^7^ cfu mL^−1^.

### 3.2. Detection Based on Metabolites Produced by Bacterial Cells as a Result of Growth

This method is based on the measurement of changes in electrical impedance of a culture medium or a reaction solution resulting from the bacterial growth. The impedance change in the medium is mainly produced by the release of ionic metabolites from the live cells, thus it could distinguish between viable and dead cells. Such a method has been developed as a rapid method that can detect bacteria within 24 h. Several commercial analytical instruments are based on this principle. These systems include the Bactometer (BioMerieux, Nuertingen, Germany), the Malthus system (Malthus Instruments Ltd., Crawley, UK), The Rapid Automated Bacterial Impedance Technique (RABIT; Don Whitley Scientific Ltd., Shipley, UK), and the Bac-Trac (Sy-Lab, Purkersdorf, Austria) [11]. However, these measurement systems are not suitable for on-the-spot applications, so many efforts have been made to minimize the instruments. Grossi *et al.* [64] developed an embedded portable biosensor system for the determination of bacterial concentration. This system is composed of an incubation chamber, containing the sample under test, and two electronic boards: one dedicated to measuring the sample electrical characteristics, the other controlling the sample temperature, fixed at a value suitable to enhance bacterial growth. Such a biosensor configuration could truly realize the miniaturization and portability. Kim *et al.* [65] proposed a plug-type, disposable electrode using a gold-coated silicon wafer, PDMS polymer, and a borosilicate glass tube to construct an impedimetric biosensor instrument. The developed biosensor could be used for *in situ* real-time monitoring of bacterial growth in a lab-scale fermentor by measuring impedance signals without the risk of introducing contamination.

Over time, much work has been done in the field of medium engineering, since the direct impedance microbiology is based on the monitoring of impedance change in the medium. The ideal medium should not only support the selective growth of the target bacteria, but also provide optimal impedance signals. For instance, one can predict that the weakly buffered media would allow a greater conductance change than the strongly buffered media. Banada *et al.* [66] used a low conductive growth medium for growth and detection of *Listeria monocytogenes* with an impedance-based microfluidics biochip detection platform. This kind of medium was suitable for growth of *Listeria monocytogenes* and the low conductive characteristic was suitable for getting greater impedance signal change due to low threshold in the variation of the impedance signal. Choi *et al.* [67] firstly attempt to use solid medium and two plane electrodes attached on two facing sides of an acryl well to fabricate an impedimetric biosensor for real-time monitoring of microorganisms. Compared to liquid medium, solid medium has advantages in that it is easy to handle and portable.

## 4. Nanomaterials

Nanomaterials, an emerging subdiscipline in chemistry have been used in impedimetric biosensors to amplify detection signal and achieve lower detection limit due to their high surface area, favorable electronic properties and electrocatalytic activity as well as good biocompatibility induced by the nanometer size and specific physicochemical characteristics [68,69]. Until now, nanomaterials [70], including metal nanoparticles, nanowires, nanorods, carbon nanotubes, and graphene, have been successfully used for constructing impedimetric biosensors for bacteria determination with enhanced analytical performance (Table 1).

In the published work, gold (Au) nanoparticles have received extensive attention in view of their easy synthesis and good stability in aqueous solution. Many efforts have been made to explore Au nanomaterials-based impedimetric biosensors. Yang *et al.* [71] reported a capacitive immunosensor for the detection of *Salmonella spp*. which was fabricated by immobilizing a Au nanoparticles monolayer onto a glassy carbon electrode and then the *Salmonella* monoclonal antibodies through physical adsorption. It was found that the Au nanoparticles can effectively improve the sensitivity and stability of the immunosensors, which can detect the *Salmonella spp*. concentrations in the range of 1.0 × 10^2^ to 1.0 × 10^5^ cfu·mL^−1^ (*R* = 0.991) with the detection limit of 1.0 × 10^2^ cfu·mL^−1^. The stability of immunosensor remained almost the same after two months storage.

In addition to Au nanoparticles, metal-oxide nanoparticles which possess high surface area and thermally stable, chemically inert, non-toxic inorganic oxide, have been also used in the development of bacteria biosensors. Huang *et al.* [69] used Fe_3_O_4_ nanoparticles to immobilize monoclonal antibodies in the construction of electrochemical impedimetric immunosensors for the rapid detection of *Campylobacter jejuni*. The Fe_3_O_4_ nanoparticles-based immunosensor showed good performance with respect to simplicity of use, fast response, wide linear range, acceptable reproducibility and long stability.

**Figure 4.**
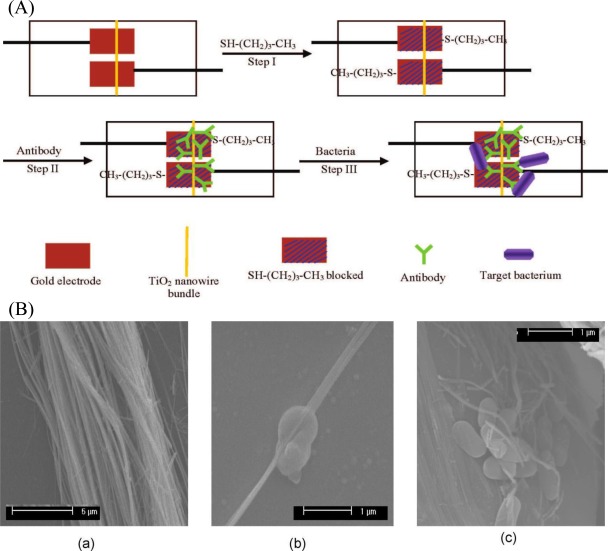
(**A**) Principle of TiO_2_ nanowire bundle microelectrode based impedance immunosensor for the detection of bacteria. (**B**) SEM micrographs of TiO_2_ nanowire bundle (**a**) before (5,000×) and (**b**,**c**) after binding with *Listeria innocua* (20,000×) [72].

In addition to nanoparticles, nanowires have been attracted much scientific interest in analytical chemistry, especially in biosensing technologies. This is due to their unique semiconductive properties associated with the nanostructures, and they are believed to be ultrasensitive in performing single molecule sensing. Wang *et al.* [72] developed a TiO_2_ nanowire bundle microelectrode based impedimetric immunosensor for rapid and sensitive detection of *Listeria monocytogenes*. TiO_2_ nanowire bundle was connected to gold microelectrodes using mask welding and then monoclonal antibodies were immobilized on the surface of a TiO_2_ nanowire bundle to specifically capture bacteria (Figure 4). Impedance changes caused by the nanowire-antibody-bacteria complex were measured and correlated to bacterial number. Since the TiO_2_ nanowires can be highly oriented on substrates or form free-standing membranes, the fabricated electrode showed a large specific surface area, good biocompatibility, good chemical and photochemical stabilities, and negligible protein denaturation. This nanowire bundle based immunosensor also exhibited a good performance that can detect as low as 10^2^ cfu·mL^−1^ of *Listeria monocytogenes* in 1 h without significant interference from other foodborne pathogens.

In recent years, reduced graphene sheets (RGSs), which are monolayers of carbon atoms packed into a dense honeycomb crystal structure, have been drawn tremendous attention from both the experimental and theoretical scientific communities. This unique nanostructure exhibits excellent electrical conductivity, mechanical strength, and chemical stability, which make it quite promising for the design of high sensitive and selective biosensors. Wan *et al.* [73] developed a RGSs-doped impedimetric immunosensor through a controllable electrodeposition method using soluble RGSs-doped CS solution for the facile and rapid detection of sulfate-reducing bacteria. They used RGSs as electron conductors to obtain good analytical performance, namely, sensitivity, selectivity, and stability, of the biosensor towards the detection of pathogen. The RGSs based immunosensor can detect the sulfate-reducing bacteria at the concentration range of 1.8 × 10^1^ to 1.8 × 10^7^ cfu·mL^−1^ and give a distinct response to sulfate-reducing bacteria without obvious response to *Vibrio angillarum*.

In addition, nanopore membrane materials such as aluminum anodized oxide nanopore membranes were used for immobilizing bioreceptors to construct impedance biosensors. Wang *et al.* [74] developed an impedimetric biosensor based on dynamic polymerase-extending hybridization for *E. coli* O157:H7 DNA detection. They immobilized ssDNA probe onto functional aluminum anodized oxide nanopore membranes. The probe strand would be extended as long as the target DNA strand, then the capability to block the ionic flow in the pores could be prominently enhanced by the double strand complex. This approach provides much lower detection limit for DNA (a few hundreds of pmol), rapid label-free and easy-to-use bacteria detection, which holds the potential for future use in various ssDNA analyses by integrated into a self-contained biochip.

Nanoscale magnetic materials have shown unique advantages that provide many exciting opportunities in bacteria detection applications. First, they can enhance the efficiency of immobilization of biofunctional molecules (e.g., antibodies, or ligands) due to their high specific surface area. Second, the nanoparticles can be manipulated by an external magnetic force, therefore, they can separate and concentrate bacteria from crude samples before impedance detection, which can detect bacteria at ultralow concentrations without time-consuming procedures and reduce the background noise ratio caused by the non-target components in the sample. Third, they improve the utilization of electrode as there is no bio-recognition biomolecule directly immobilized on the electrode surface. Due to the above advantages, many researchers have used the bio-recognition elements immobilizing on magnetic beads to separate and concentrate bacteria in samples, and then the combination complexes of the biofunctional magnetic nanoparticles and bacteria were measured as an impedance signal [75–79].

An example can be found in the determination of *Salmonella typhimurium*. Anti-*Salmonella* antibodies were coated with immunomagnetic beads to separate *Salmonella typhimurium* from samples. Then the concentrated sample was spread on the surface of electrodes to detect impedance signal over a range of frequency. A linear relationship between the detection time and the logarithmic value of the initial cell number was found in the *Salmonella* cell number ranging from 10^1^ to 10^6^ cfu mL^−1^ [80]. Madhukar *et al.* [81] developed an impedance biosensor based on IDAM coupled with magnetic nanoparticles-antibody conjugates (MNAC) for rapid and specific detection of *E. coli* O157:H7 in ground beef samples. MNAC were prepared by immobilizing biotin-labeled polyclonal goat anti-*E. coli* antibodies onto streptavidin-coated magnetic nanoparticles, which were used to separate and concentrate *E. coli* O157:H7 from ground beef samples. Magnitude of impedance and phase angle were measured in a frequency range of 10 Hz to 1 MHz in the presence of 0.1 M mannitol solution. The equivalent circuit analysis showed that bulk resistance and double layer capacitance were responsible for the impedance change caused by the presence of *E. coli* O157:H7 on the surface of IDAM. The lowest detection limits of this biosensor for detection of *E. coli* O157:H7 in pure culture and ground beef samples were 7.4 × 10^4^ and 8.0 × 10^5^ cfu·mL^−1^.

## 5. Microfluidics Techniques

**Figure 5.**
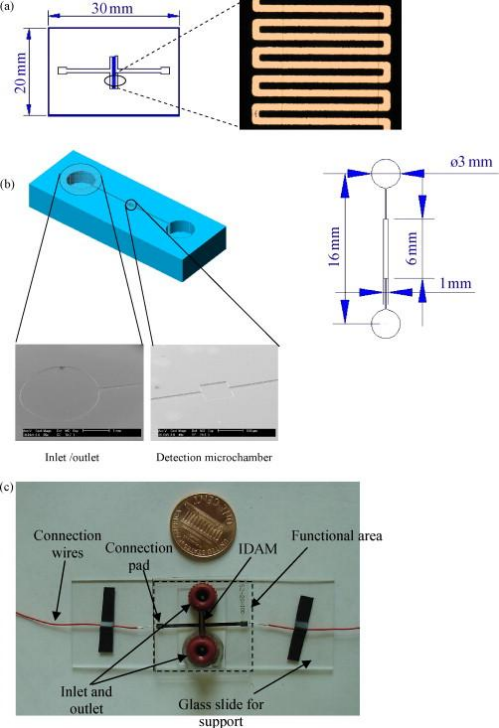
(**a**) IDAM chip with gold microelectrodes on a glass wafer, (**b**) a microchannel with a detection microchamber, and inlet and outlet channels, and (**c**) an assembled microfluidic flow cell with embedded IDAM and connection wires [84].

Besides nanomaterials, microfluidics techniques are a good strategy for improving the performance of impedimetric bacteria biosensors [86–94]. Microfluidics techniques in general seek to improve analytical performance by reducing the consumption of reagents, decreasing the analysis time, increasing reliability and sensitivity through automation, and integrating multiple processes in a single device. These features are particularly suitable for hand-held impedance biosensors for bacteria detection [95]. Varshney *et al.* [84] integrated a microfluidics flow cell with embedded gold IDAM into an impedance biosensor to rapidly detect pathogenic bacteria in ground beef samples. The flow cell consisting of a detection microchamber and inlet and outlet microchannels was fabricated by binding an IDAM chip to a poly (dimethylsiloxane) (PDMS) microchannel (Figure 5). The detection microchamber with a dimension of 6 mm × 0.5 mm × 0.02 mm and a volume of 60 nL was used to collect bacterial cells in the active layer above the microelectrode for sensitive impedance change. Antibody coated magnetic nanoparticles were used to specifically separate and concentrate the target bacteria and then the biomolecule functional magnetic nanoparticles-bacteria complexes were injected into microfluidic cell to detect the impedance change. Using the microfluidic system, the limit of detection has been improved an order of magnitude as low as 1.6 × 10^2^ and 1.2 × 10^3^ cfu·mL^−1^ of *E. coli* O157:H7 cells present in pure culture and ground beef sample, respectively. Tan *et al.* [82] devised a PDMS microfluidic immunosensor integrated with specific antibody immobilized alumina nanoporous membrane for rapid detection of foodborne pathogens *E. coli* O157:H7 and *Staphylococcus aureus* with EIS. When the target bacteria were injected into the chamber to bind antibody, the electrolyte current will be blocked which can be monitored by the impedance spectrum. This microfluidic immunosensor based on nanoporous membrane impedance spectrum could achieve rapid bacteria detection within 2 h with a high sensitivity of 10^2^ cfu mL^−1^.

The microfluidic biochip system has been used to effectively improve the detection limit and reduce detection time of impedance biosensor for bacteria detection by confining a few live bacterial cells into a small volume on the order of nano-to pico-liters. Gomez and coworkers [96] were the first to fabricate integrated silicon-based microfluidic biochips for impedance detection of microbial metabolism. The impedance microbiology-on-a chip contained two sets of interdigitated microelectrodes. One set was for dielectrophoretically capturing bacterial cells from the flow into the small chamber, and the other set was for monitoring the impedance change when bacterial cells grew in the chamber. The design concept was to use DEP to deviate the bacterial cells from a main channel into a small channel that led the cells into a measurement chamber which had a volume of 400 pL. This on-chip impedance microbiology has achieved a detection time of 1 h for a sample with a starting concentration of 10^4^ cfu·mL^−1^. A similar microfluidic system has been developed to concentrate bacteria. Yang *et al.* [36] developed a microfluidic system with multiple functions, including concentration of bacteria using DEP and selective capture using antibody recognition, resulting in a high capture efficiency of bacterial cells. The device consisted of an array of oxide covered interdigitated electrodes on a flat silicon substrate and a ∼16 mm high and ∼260 mm wide micro-channel within a PDMS cover. The impedimetric biosensor that combined DEPIM offered advantages inherited from both DEP and antibody recognition, including increasing antibody capture efficiency and decreasing binding time of bacteria and antibody.

In addition, low cost and disposability is another trend for microfluidics biochip development. Gottschamel *et al.* [88] developed a disposable microfluidics biochip for multiparameter *Candida albicans* population measurements, which can monitor *Candida albicans* growth rates and metabolic activities by simultaneous bioimpedance spectroscopy and amperometric measurements. Zhu *et al.* [92] used fluidic electrodes to fabricate a microfluidics device for detecting bacterial cells in deionized water suspensions with a detection limit of 10^3^ cfu·mL^−1^. KCl solution was utilized as both sheath flow and fluidic electrodes, and the bacterial suspension was squeezed to form three flowing layers with different conductivities on a microfluidics chip. An impedance analyzer was connected with the KCl solution through two Ag/AgCl wires to apply an AC voltage to fluidic layers within a certain frequency for impedance measurements. Compared with traditional metal electrode, the use of fluidic electrodes can effective decrease the cost for fabrication of a microfluidics biochip.

## 6. Conclusions

Impedimetric biosensors have been used to monitor foodborne pathogenic bacteria for many years. Compared to the other methods, it has several main advantages as follows:

– They are label-free, which simplifies the assembly process and lowers the cost.
– They are rapid and the detection time is generally less than 30 min.
– Realization of the impedance device miniaturization, which have been proved to be very successful in maximizing the impedance signal, minimizing the volume of testing sample, increasing sensitivity, and saving assay time.
– They can reach detection limits as low as those of SPR and ELISA. After combination of nanoparticles or with microfluidic techniques, they can achieve a lower detection limit than standard immunoassays.
– They are reproducible when the bio-recognition elements are immobilized on the electrode using strong chemical bonds such as SAM immobilization mehod, which reduces the cost of use.

Although the impedimetric biosensors have many advantages, they still have some limits. After two decades of research efforts and hundreds of publications, no product based on impedance-based biosensors has enjoyed widespread commercial success. Therefore, further efforts should be devoted to developing commercial products in the area of impedimetric biosensors for foodborne pathogenic bacteria detection, which will require improved stability, reduced volume, increased sensitivity and lowered costs.

## Figures and Tables

**Table 1.** Comprehensive list of nanomaterials based impedimetric biosensors for detection of foodborne pathogenic bacteria.

**Nanomaterials**	**Microorganism**	**Electrode**	**Detection range (cfu·mL^−1^)**	**Reference**
Au NPs	Sulfate-reducing bacteria	foam Ni electrode	2.1 × 10^1^–2.1 × 10^7^	[28]
Fe_3_O_4_ NPs	*Campylobacter jejuni*	GCE	1.0 × 10^3^–1.0 × 10^7^	[69]
Au NPs	*Salmonella Spp.*	GCE	1.0 × 10^2^–1.0 × 10^5^	[71]
TiO_2_ nanowire bundle	*Listeria monocytogenes*	Au microelectrodes	10^2^–10^7^	[72]
reduced graphene sheets	Sulfate-reducing bacteria	GCE	1.8 × 10^1^–1.8 × 10^7^	[73]
aluminum anodized oxide (AAO) nanopore membranes	*E. coli* O157:H7	Au electrode	–	[74]
alumina nanoporous membrane	*E. coli* O157:H7	Platinum electrode	10^2^–10^7^	[82]
carbon nanofiber (CNF) nanoelectrode array (NEA)	*E. coli*	ITO	–	[83]
magnetic nanoparticles	*E. coli* O157:H7	IDAM	pure culture 7.4 × 10^4^–7.4 × 10^7^ beef sample 8.0 × 10^5^–8.0 × 10^7^	[81]
magnetic nanoparticles	*E. coli* O157:H7	IDAM with microfluidic flow cell	pure culture 1.6 × 10^2^–1.6 × 10^7^ beef sample 1.2 × 10^3^–1.2 × 10^7^	[84]
magnetic nanoparticles	*E. coli*	Pt plate electrode	10–10^4^	[85]

ITO: indium-tin oxide; GCE: glassy carbon electrode; Au NPs: gold nanoparticles.
